# An intronic variant in *Ferredoxin Reductase (FDXR)* creates a cryptic exon in Quarter Horses with Equine Juvenile Spinocerebellar Ataxia

**DOI:** 10.1371/journal.pgen.1012158

**Published:** 2026-05-20

**Authors:** Briana N. Brown, Anna R. Dahlgren, Sharmila Ghosh, Blythe Durbin-Johnson, Andrew Willis, Cassandra Olivas, Daniel York, Robert Grahn, Rebecca R. Bellone, Gino A. Cortopassi, Andrew D. Miller, C. Titus Brown, Kevin Woolard, Carrie J. Finno

**Affiliations:** 1 Department of Population Health and Reproduction, School of Veterinary Medicine, University of California-Davis, Davis, California, United States of America; 2 Bioinformatics Core Facility, Genome Center, University of California-Davis, Davis, California, United States of America; 3 Weatherford Equine Medical Center, Weatherford, Texas, United States of America; 4 Department of Surgical and Radiological Sciences, School of Veterinary Medicine, University of California-Davis, Davis, California, United States of America; 5 Veterinary Genetics Laboratory, School of Veterinary Medicine, University of California-Davis, Davis, California, United States of America; 6 Department of Molecular Biosciences, School of Veterinary Medicine, University of California-Davis, Davis, California, United States of America; 7 Department of Biomedical Sciences, Section of Anatomic Pathology, College of Veterinary Medicine, Cornell University, Ithaca, New York, United States of America; 8 Department of Anatomic Pathology, School of Veterinary Medicine, University of California-Davis, Davis, California, United States of America; INCIA: Institut de Neurosciences Cognitives et Integratives d’Aquitaine, FRANCE

## Abstract

Equine Juvenile Spinocerebellar Ataxia (EJSCA) is a novel autosomal recessive neurologic disease in Quarter Horses. Affected foals display a progressive proprioceptive ataxia by 1–5 weeks of age, leading to recumbency and necessitating euthanasia. Whole genome sequencing was performed on 7 EJSCA cases and unaffected horses that included 4 obligate carriers, 4 unaffected half or full-siblings, and 28 unrelated, unaffected control Quarter Horses. An 82 kb region of association was identified (EquCab3.0, chr11: 6963986–7045999), containing 9 candidate SNPs across four genes (*FADS6, FDXR, GRIN2C* and *TMEM104*). Decreased *FDXR* mRNA expression and a cryptic exon was identified in spinal cord tissue from EJSCA cases via RNA-sequencing. One of the 9 associated SNPs (*FDXR-203* c.177 + 1778G > C) was the eighth base pair of this cryptic exon. Affected foals were all homozygous for the variant. Protein concentrations of FDXR were lower in EJSCA cases in spinal cord and liver compared to unaffected controls. The *FDXR-203* c.177 + 1778G > C mutation represents the first non-coding neurological genetic variant in horses. Additionally, this is the first genetic cause of a degenerative axonopathy in the horse and a spontaneous disease model to study FDXR pathology in humans.

## Author summary

The American Quarter Horse is the most popular horse breed in North America. A rapidly progressive spinocerebellar ataxia, termed equine juvenile spinocerebellar ataxia (EJSCA), was first identified in Quarter Horse foals in 2020. EJSCA was shown to be inherited as an autosomal recessive trait. Whole-genome sequencing on 7 ESJCA cases and RNA-sequencing of spinal cord identified an intronic variant in *Ferredoxin reductase* (*FDXR*) creating a cryptic third exon in affected foals*.* Four additional affected foals were subsequently genotyped and were all homozygous for the intronic variant. Decreased transcript and protein expression of FDXR was observed in spinal cord and liver of affected animals. The *FDXR-203* c.177 + 1778G > C mutation represents the first disease-causing genetic variant for an inherited degenerative axonopathy in the horse and a spontaneous disease model to study FDXR pathology in humans.

## Introduction

In 2020, the first identified case of equine juvenile spinocerebellar ataxia (EJSCA) in the American Quarter Horse (QH) was recognized [1]. Over the next 4 years, an additional eleven cases were identified. All foals were presented between 1–5 weeks of age with an acute history of neurological deficits, with the pelvic limbs more severely affected. The spinal ataxia rapidly progressed to include paraparesis, resulting in recumbency within 0–18 days and necessitating euthanasia [1]. Postmortem lesions were confined to histologic evidence of dilated myelin sheaths and digestion chambers throughout the dorsal spinocerebellar tract of entire spinal cord, but most severe in the cervicothoracic region. Some foals had additional lesions within the fasciculus cuneatus and ventromedial tracts [1]. The pedigree analysis of cases supported an autosomal recessive mode of inheritance, with common ancestors tracing back within 4–6 generations on both sides of the pedigree [1].

Inherited defects that result in a spinocerebellar ataxia have been reported in dogs [2–9], cattle [10–15] and sheep [16–18]. In horses, a distinct spinocerebellar ataxia, termed equine neuroaxonal dystrophy/ degenerative myelopathy, has been described but horses develop less severe clinical signs later in life (typically 1–3 years of age) [19]. Despite several intensive genetic investigations, there is currently no genetic mutation identified for eNAD/EDM. Further, EJSCA is a clinically and histologically distinct disorder [1].

Based on the apparent familial basis for EJSCA, we aimed to identify putative genetic variants associated with the phenotype. We performed whole-genome sequencing in seven affected QH foals with EJSCA and explored differences in gene expression within our candidate region in spinal cord tissue. Segregating coding variants were excluded, and we identified an intronic variant, resulting in a cryptic exon in *Ferredoxin reductase* (*FDXR* c.177 + 1778G > C), that significantly associated with the EJSCA phenotype. PromethION Nanopore sequencing was used to clearly define the boundaries of the cryptic exon and to determine that inclusion of the in-frame cryptic exon led to a premature stop codon. Decreased FDXR transcript and protein expression supported loss-of-function in the spinal cord and liver of affected foals, which aligned with clinical findings.

## Results

### Animals

Clinicopathologic findings of foals affected with EJSCA were previously described [1]. Briefly, the study included n = 11 confirmed EJSCA foals (n = 9 fillies and n = 2 colts, which were included in the previous report [1]) that appeared healthy at birth but developed an acute onset of neurologic deficits within the first months of life (S1 Table). Deficits consisted of a spinocerebellar ataxia of all four limbs, with mild asymmetric paraparesis in some cases. Clinicopathologic findings included elevated gamma-glutamyl transferase (GGT) and hyperglycemia on biochemical profiles, with normal muscle enzymes. All foals rapidly progressed to recumbency and were euthanized. Postmortem evaluation identified dilated myelin sheaths with digestion chambers throughout the entire spinal cord but most severe in the dorsal spinocerebellar tracts of the cervicothoracic region [1]. Control horses were considered healthy if they had lived past six months of age with no evidence of ataxia.

### Whole-Genome Genome-Wide Association Study (WGS-GWAS)

**Fig 1:**
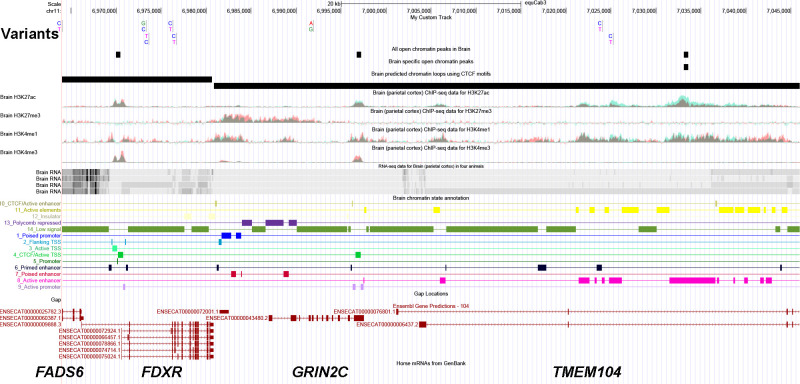
Whole genome association study identified nine homozygous SNPs across the genes *FADS6*, *FDXR*, *GRIN2C*, and *TMEM104* that were only present in EJSCA foals. Whole genome association testing identified an 82 kb candidate region of interest (p = 2.9 x 10^-12^) that was homozygous in all affected foals. After filtering by effect (‘MODERATE’ and ‘HIGH’) and screening in public databases (https://ncbi.nlm.nih.gov/subs/sra/ and [20]), nine homozygous variants across the genes *Fatty acid desaturase 6 (FADS6*, 1 synonymous SNV*), Ferredoxin reductase (FDXR*, 4 intronic SNVs), *Glutamate ionotropic receptor NMDA type subunit 2C (GRIN2C*, 1 intronic SNV) and *Transmembrane protein 104 (TMEM104* (3 intronic SNVs) were only present in EJSCA foals. Top tracks represent equine FAANG data tracks.

A whole-genome association genome-wide association (WGS-GWAS) study was performed to investigate the genetic cause of EJSCA. Significantly associated variants were identified, and their effects predicted using the whole-genome sequence of n = 7 EJSCA foals, n = 3 carrier dams, n = 1 carrier sire, n = 3 unaffected half-siblings, n = 1 unaffected full-sibling and n = 28 unrelated control Quarter Horses (S1 Table). Seventy-seven variants were associated with EJSCA at p < 1 x 10^-11^, and all were homozygous in EJSCA foals and heterozygous in the carriers. The 77 associated variants were in an 82 kb region on chr11 from 6963986-7045999 bp (S2 Table). The variants within this region were further filtered by effect (‘MODERATE’ and ‘HIGH’) and screened in public databases (https://ncbi.nlm.nih.gov/subs/sra/ and [20]). No ‘HIGH’ effect nonsense variants were identified. All ‘MODERATE’ effect variants were found in other breeds with no reported cases of this neurological condition (S2 Table). Of the remaining single nucleotide variants (SNVs), only 9 were not identified in any other breed (Table 1). These variants spanned four genes: *Fatty acid desaturase 6 (FADS6*, 1 synonymous*), Ferredoxin reductase (FDXR*, 4 intronic SNVs), *Glutamate ionotropic receptor NMDA type subunit 2C (GRIN2C*, 1 intronic SNV) and *Transmembrane protein 104 (TMEM104* (3 intronic SNVs) (Fig 1). All genes are expressed in human spinal cord RNA (https://www.proteinatlas.org/) [21]. *FADS6*, *FDXR* and *GRIN2C* are expressed at the RNA level in equine spinal cord and cerebellum, but *TMEM104* is not [22]. Scores were determined for each orthologous human variant using the 100-vertebrate score by phastCons (https://genome.ucsc.edu/). All conservation scores were 0, except for chr11:6973334, which was 0.05.

**Table 1:** Nine putative genetic variants for EJSCA based on whole-genome sequencing-genome-wide association study. CHR = chromosome, POS = position, REF = reference allele, ALT = alternate allele, POS, REF, and ALT are according to the Equcab3.0 assembly. Impact was defined using SNPSift [23].

CHR	POS	REF	ALT	SnpEff Putative Effect	*P*_allelic_	Impact
11	6963986	C	T	*FADS6* synonymous (*FADS6* c.579C > T, p.Asn193Asn)	2.99 x 10^–12^	LOW
11	6973334	G	C	*FDXR* intronic	2.99 x 10^–12^	MODIFIER
11	6973640	T	C	*FDXR* intronic	2.99 x 10^–12^	MODIFIER
11	6976294	C	T	*FDXR* intronic	2.99 x 10^–12^	MODIFIER
11	6976720	C	T	*FDXR* intronic	2.99 x 10^–12^	MODIFIER
11	6991960	A	G	*GRIN2C* intronic	2.99 x 10^–12^	MODIFIER
11	7024027	C	T	*TMEM104* intronic	2.99 x 10^–12^	MODIFIER
11	7025248	C	T	*TMEM104* intronic	2.99 x 10^–12^	MODIFIER
11	7045999	C	G	*TMEM104* intronic	2.99 x 10^–12^	MODIFIER

### mRNA-sequencing

**Fig 2:**
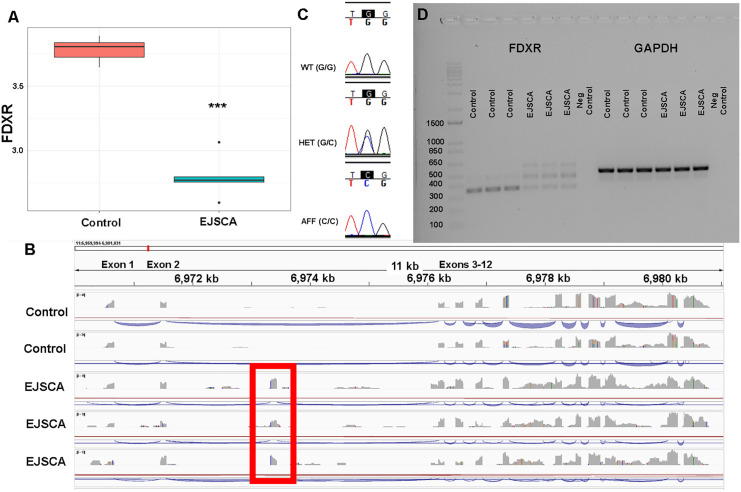
FDXR investigation and functional validation. **(A)** Bulk mRNA sequencing of spinal cord tissue performed on n = 5 EJSCA cases and n = 6 unaffected age-matched controls identified significantly decreased *FDXR* expression in EJSCA foals at P_unaadjusted_ = 0.0008). **(B)** Mapped mRNA reads over *FDXR* identified a cryptic exon (indicated in red box) in intron 2 in EJSCA foals, corresponding to one of the *FDXR* deep intronic variants identified in the WGS-WGAS (chr11:6973334, G/C). **(C)** Representative chromatograms from Sanger sequencing of n = 11 EJSCA foals, n = 12 dams, n = 4 sires, n = 11 siblings, and n = 8 unrelated healthy control horses confirmed perfect segregation of the c.177 + 1778G > C variant. **(D)** RT-PCR of *FDXR* exons 2-5 identified three faint amplicons in EJSCA foals (n = 3) and two amplicons (one bold, one faint) in unrelated control horses (n = 3). *GAPDH* is the positive control. The ladder is on the left of the gel, indicating band sizes from 100 bp to 1500 bp.

To quantify gene expression across the four genes of interest in the candidate region (*FADS6*, *FDXR, GRIN2C* and *TMEM104*), mRNA-sequencing on spinal cord tissue was performed on n = 5 EJSCA cases and n = 6 unaffected age-matched controls (S1 Table). There were no genes that were significantly different at P_FDR_ < 0.05. However, of the four positional candidate genes (*FADS6*, *FDXR*, *GRIN2C* and *TMEM104*), only *FDXR* was significantly decreased in EJSCA foals as compared to healthy foals at P_unadjusted_<0.01 (p = 0.0008) (Fig 2A). Mapped RNA-seq reads were then visualized for *FDXR*, and a cryptic exon was identified in intron 2, which contained the intronic variant identified at chr11:6973334 (G/C) (Fig 2B).

### qRT-PCR Validation of *FDXR* expression

To validate the decreased expression of *FDXR* observed in spinal cord from EJSCA foals, qRT-PCR was performed in n = 3 EJSCA foals and n = 3 age-matched unrelated healthy control foals. *FDXR* expression was decreased in EJSCA affected foals (fold-change = 0.44, p = 0.02).

### Variant Validation via Sanger Sequencing

DNA from n = 11 EJSCA foals, n = 12 dams, n = 4 sires, n = 11 siblings, and n = 8 unrelated healthy control horses underwent Sanger sequencing to confirm the putative *FDXR* variant detected from whole genome sequencing data (c.177 + 1778G > C). All EJSCA foals were homozygous for the variant while all dams and sires were heterozygous. Unrelated control samples were homozygous wildtype (Fig 2C). An additional 1,060 available presumed unaffected Quarter Horses were randomly selected from the biorepository at the UC Davis Veterinary Genetics Laboratory and genotyped for this variant. From the 1,060 horses, 25 were heterozygous and the remainder of the horses were homozygous wildtype. The allele frequency for the *FDXR* variant was calculated from this sample set for the Quarter Horse population (*q* = 0.012). An additional 181 horses across 26 breeds from the University of California Veterinary Genetics Laboratory were screened and no other carriers were identified in other breeds (S3 Table).

### *FDXR* c.177 + 1778G > C functional validation

Since the putative variant for EJSCA was deep within an intron, 1778 bp away from exon 2, and the RNA-sequencing data did not clearly define the boundaries of the cryptic exon, we pursued reverse-transcriptase PCR (RT-PCR) in spinal cord, liver and gluteal muscle from EJSCA affected foals and age-matched controls. Primers flanking exons 2 and 3 of *FDXR*-201, as annotated by Ensembl EquCab3.0, (https://useast.ensembl.org/Equus_caballus/Info/Index), amplified two PCR products, one prominent band and one band with less intensity in control horses (~350 bp and ~480 bp, respectively, Fig 2D). Three products were observed in EJSCA foals (~350, 480 and 650 bp, Fig 2D).

**Fig 3:**
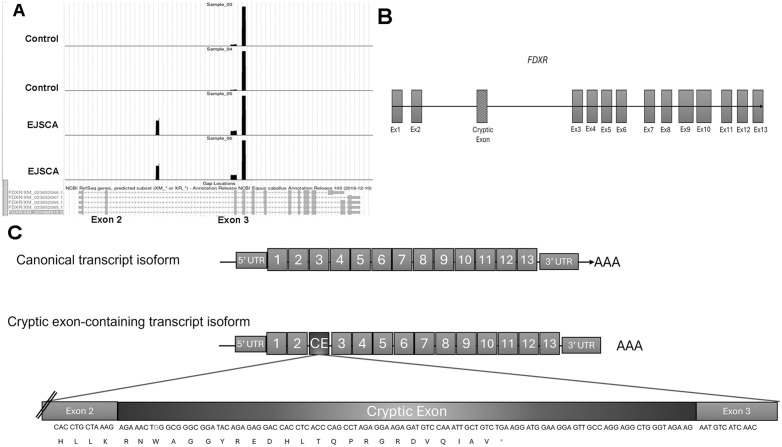
Long-read PromethION Nanopore sequencing of *FDXR* cDNA amplicons from spinal cord tissue. **(A)** Intronic *FDXR* reads were identified in intron 2 only in EJSCA samples, consistent with cryptic exon localization. **(B)** The PromethION Nanopore sequencing allowed for accurate detection of the cryptic exon boundaries. **(C)** When the cryptic exon is transcribed, the additional amino acids are in-frame but result in a premature stop codon (“TGA”) after 24 amino acids.

Long-read PromethION Nanopore sequencing of the resulting RT-PCR products from spinal cord tissue was performed in n = 2 EJSCA foals and n = 4 controls to define these fragments. A consensus sequence was identified, and two clusters (Cluster 24 and 26, both 306 bp on average) demonstrated allele sharing across all six samples (EJSCA and controls). The two other clusters (Cluster 0, 398 bp and Cluster 144, 541 bp) were quantified at 30-2880x only in EJSCA foals (Fig 3A and S4 Table). Clusters 0 and 144 each contained sequences that aligned over the cryptic exon that had been identified with the RNA-sequencing data. Cluster 144 also contained a longer 5’ end of canonical exon 3. Within both clusters 0 and 144, the G > C substitution was evident at 8 bp (Cluster 0) or 29 bp (Cluster 144) from the start of the reads in EJSCA foals only. Based on the PromethION Nanopore sequencing, the boundaries of the cryptic exon were clearly defined **(**Fig 3B) and the resulting amino acid sequence determined (Fig 3C). Inclusion of the in-frame cryptic exon led to a premature stop codon after 24 amino acids (Fig 3C).

**Fig 4:**
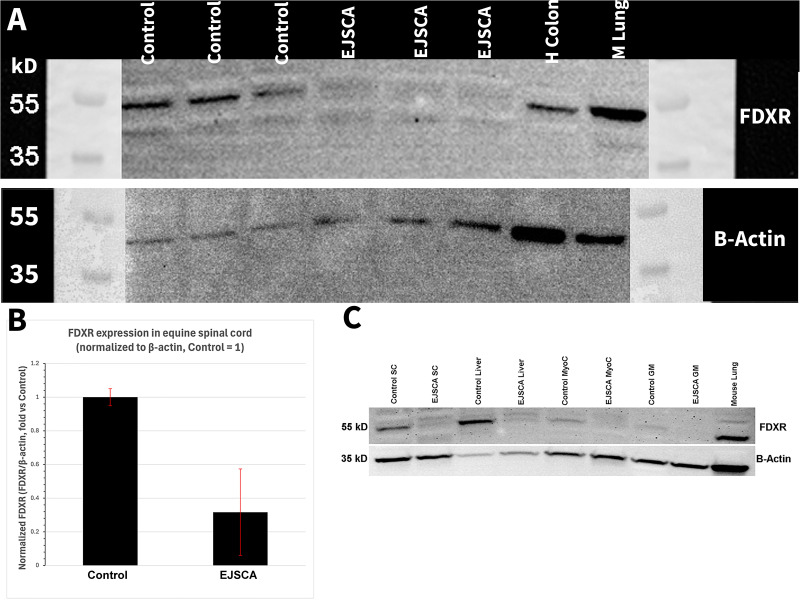
Western blot from tissues of EJSCA foals and age-matched unrelated control foals. **(A)** FDXR protein expression was decreased in spinal cord of FDXR foals (n = 3 biologic replicates) as compared to age-matched unrelated control foals. **(B)** Quantification of decreased spinal cord expression of FDXR. **(C)** FDXR protein expression was decreased in all EJSCA foal tissues but is particularly evident in spinal cord and liver. Overall expression was decreased in myocardium and gluteal muscle. Beta-actin was the loading control. Biological replicates and original gel images are included in S1 Fig. SC = spinal cord, MyoC = myocardium, GM = Gluteal muscle, H = human, M = mouse.

Western blotting of spinal cord demonstrated decreased FDXR protein expression in EJSCA foals compared to unaffected control horses (p = 0.04, Fig 4A and 4B). Additionally, decreased expression was observed in myocardium and gluteal muscle from EJSCA foals; however, the overall expression in these tissues across all horses was lower (Fig 4C). Biological replicates of western blotting from spinal cord and original gel images are included in S1A, S1B, S1C, S1D, and S1E Fig.

## Discussion

There are currently six well-characterized disease variants in the American Quarter horse that are recommended for testing as part of the American Quarter Horse Association Health Panel. These include glycogen branching enzyme deficiency (GBED), hereditary equine regional dermal asthenia (HERDA), hyperkalemic periodic paralysis (HYPP), malignant hyperthermia (MH), polysaccharide storage myopathy Type 1 (PSSM1) and myosin-heavy chain myopathy (MYHM). Testing for this panel is required by the breed association for stallions before resulting foals are registered. The variants for these six diseases are all coding in nature, including missense (HERDA, HYPP, MH, PSSM1, MYHM) and nonsense (GBED) mutations. The *FDXR* c.177 + 1778G > C mutation identified in this study represents the first intronic variant that results in a severe fatal neurologic disease in Quarter Horses. Additionally, this *FDXR* variant represents the first identified cause for an inherited degenerative axonopathy in the horse and a spontaneous disease model to study FDXR pathology in humans.

While horses are not a common model for studying human diseases, the discovery of a missense variant in *Myosin heavy chain 1* (*MYH1*) [24] in horses led to the prioritization of *MYH1* as a candidate gene for recurrent rhabdomyolysis in humans [25]. Additionally, horses suffering from malignant hyperthermia have a mutation in the *Ryanodine receptor* 1 (*RYR1*) [26], like humans [27]. A pathogenic variant in the ER-associated degradation pathway protein SEL1L was identified in a rare platelet disease in horses [28], which spurred a cross-species approach that has established *SEL1L* variants as candidates for human bleeding disorders [29]. Thus, leveraging genetic findings in the horse can lead to new discoveries with strong translational relevance.

In humans, *FDXR* mutations are associated with mitochondriopathies defined by decreased electron transport chain function, increased reactive oxygen species and iron overload [30]. The first link between *FDXR* and mitochondrial disease in humans was published in 2017, where recessive mutations in *FDXR* resulted in optic atrophy, ataxia and hypotonia in a cohort of individuals [31]. Functional studies in cells from these patients showed reduced FDXR activity and mitochondrial overload. That same year, eight children with auditory neuropathy and bilateral defects of the optic nerves were identified with recessive *FDXR* mutations [32]. Since then, the disease has been renamed FDXR-related mitochondriopathy (FRM) and symptoms were expanded to include movement disorders and global developmental delay [33]. All the pathogenic variants described to date in humans are missense, in frame deletions, or nonsense variants (https://www.ncbi.nlm.nih.gov/clinvar), with no splice site variants identified to date. Foals with EJSCA did not appear to have visual or auditory deficits, but electroretinography and brainstem auditory-evoked potential testing was not performed. Optic nerves are not routinely collected during veterinary necropsies, but should be evaluated in any future cases of this disorder

Deep intronic mutations can disrupt normal splicing patterns by creating new splice sites, altering splicing enhancers or silencers or interfering with canonical splice sites. Deleterious DNA variants that are more than 100 bp away from exon-intron junctions commonly lead to pseudo-exon inclusion [34]. With EJSCA, the deep intronic *FDXR* c.177 + 1778G > C mutation creates a cryptic splice site, which likely activates nonsense-mediated decay, as evidenced by reduced mRNA expression and the fainter RT-PCR bands, as well as reduced protein observed. The advantage of performing whole-genome sequencing, versus whole exome sequencing, was clearly highlighted in this study, as an exome approach would have failed to detect this variant. Pairing our whole-genome results with reads obtained from RNA-sequencing was critical in identifying a putative functional intronic variant in *FDXR*.

While EquCab3.0 has undoubtedly improved the reference assembly for the horse, annotation of specific isoforms still relies primarily on in silico predictions through Ensembl. There are six transcripts of *FDXR* annotated in Ensembl (FDXR-201,202,203,204,205 and 206), with FDXR-201 labeled as the “canonical” isoform. Using the NCBI annotation, which relies more on RNA-seq data to identify coding regions, there are five transcripts (XM_023652066.1 or variant X4, XM_023652067.1 or variant X5, XM_023652064.1 or variant X1, XM_023652065.1 or variant X2 and XM_001492615.6 or variant X2). Using the Ensembl annotation, the main variation between isoforms occurs in the 5’ UTR region and 3’ to exon 3, with alternative splicing annotated between exons 3 and 4, 4 and 5 and after exon 10. In NCBI, the variation is 3’ to exon 10, with alternative splicing denoted between exons 11, 12 and 13. Notably, none of these isoforms have coding sequence predicted between canonical exons 2 and 3, and none of these isoforms support a longer 5’ end to exon 3 that was noted in our EJSCA foals (as shown by PromethION long-read sequencing results). Within the human genome, there are eight isoforms and none include an additional exon between canonical exons 2 and 3. Thus, this disease in the horse is associated with a novel splice variant of *FDXR*.

Within NCBI, two proteins are predicted for FDXR in the horse: XP_070082099.1 (isoform 1, 1918 nucleotides) and XP_001492665.3 (isoform 2, 1912 nucleotides), which only differ by 6 nucleotides. The protein sequence for FDXR is well-conserved between horses and humans (89% identity, 100% query cover, E value = 0.0 via BLAST search (https://blast.ncbi.nlm.nih.gov). Loss-of-function variants in *FDXR* are associated with mitochondrial iron overload, and this accumulation results in reactive oxygen species accrual and ferroptosis [30]. Recent evidence suggests that *Nuclear factor erythroid 2-related factor 2 (NRF2)*, a transcription factor that is activated during oxidative stress, is downregulated in FDXR-related mitochondrial disease in humans [35].

Since we were unable to amplify cDNA from the region between canonical exons 2 and 3 of *FDXR* using standard nested PCR and Sanger sequencing techniques, we elected to leverage cDNA Nanopore long-read sequencing technology to sequence each fragment. Major isoforms of genes have been readily identified in human cell lines using Nanopore long-read sequencing protocols [36]. Gene expression estimates from Nanopore long-read RNA-seq data showed the lowest estimation error overall when compared to various RNA-seq protocols [36]. In our study, this long-read sequencing technology provided detailed insight into the alternative splicing that resulted from the *FDXR-203* c.177 + 1778G > C variant. This study represents the first use of Nanopore long-read sequencing technologies in the horse to identify novel splice events resulting from intronic variants.

Clinically, foals with EJSCA are presented with a spinocerebellar ataxia and clinicopathologic evidence of liver injury, as evidenced by high GGT concentrations. Muscle enzymes (creatine kinase [CK] and aspartate aminotransferase [AST]) are within normal limits on these foals. This clinical presentation is reflected in the tissue-specific expression that we identified for FDXR. Expression was highest, at both the RNA and protein level, in spinal cord and liver and lower in gluteal muscle and myocardium. In the human protein atlas (https://www.proteinatlas.org), FDXR expression is noted to be highest in adrenal gland (RNA and protein levels), testis and epididymis (protein only), and fallopian tube and ovary (protein only). Within the nervous system, expression is quantifiable, but much lower, in human cerebellum and spinal cord at the RNA level only. We were able to leverage the functional atlas of animal genomes (FAANG) dataset that was created in the horse to evaluate equine-specific tissue patterns of gene expression and regulation (https://www.ebi.ac.uk/ena/data/view/ERA1487553) [22]. *FDXR* was expressed at the RNA level in equine spinal cord and cerebellum, with no functional elements (promoters, enhancers, silencers) predicted at the site of the EJSCA variant (*FDXR-203* c.177 + 1778G > C). Since *FDXR* expression was identified in equine spinal cord and cerebellum, the FAANG dataset provided species-specific gene expression information that was advantageous to this investigation.

It is worthwhile noting that, of the 9 putative variants (Table 1), the *FDXR-203* c.177 + 1778G > C variant was the only one that did not have a conservation score of 0. While variants that disrupt canonical splice site elements often adhere to a highly conserved consensus sequence, de novo mutations that create cryptic exons do not [37]. Thus, while using conservation scores to prioritize putative variants remains an important tool, variants should not be excluded as candidates simply due to low conservation scores.

Limitations of this study include the small sample sizing of EJSCA affected foals, limited frozen spinal cord tissue for extensive qRT-PCR validation of all cases, inclusion of only female cases in RNA-sequencing and qualitative analysis of FDXR protein in the liver. With the family-based cohort, a WGS-GWAS was not the ideal model for performing association testing and either family-based association testing or homozygosity mapping could have been pursued, with the likely autosomal recessive mode of inheritance. We did not have a large multi-generational family to track disease segregation and, with the high background inbreeding in this family of horses, homozygosity mapping could have resulted in spurious associations.

In summary, this study identified a putative variant associated with EJSCA in the American Quarter Horse (*FDXR-203* c.177 + 1778G > C). Supporting evidence for causality include: (1) this variant is the 8^th^ base pair of a cryptic exon that occurs only in EJSCA affected foals, (2) there is an overall decrease in *FDXR* expression in spinal cord of EJSCA foals, (3) perfect genotyping concordance was observed between the *FDXR-203* c.177 + 1778G > C variant and the EJSCA phenotype and (4) comparative knowledge of a similar phenotype in humans with genetic mutations in *FDXR*. As such, to avoid producing affected offspring, utilization of genetic testing results for the *FDXR*-203 c.177 + 1778G > C variant is recommended for any Quarter Horse breeding program. The overall low allele frequency across the QH breed and evidence of all affected foals tracing back to one prominent sire within 6 generations [1] suggests that this is a relatively new genetic variant. Notably, identification of this deep intronic variant was only possible with whole-genome sequencing. This *FDXR* variant represents the first to explain an inherited degenerative axonopathy in the horse and a spontaneous disease model to study FDXR pathology in humans.

## Materials and methods

### Ethics statement

All procedures were approved by the University of California-Davis Institutional Animal Care and Use Committee (#23015) and carried out in accordance with guidelines and regulations. Written consent from the owners was obtained for all sample collections.

### Animals

Clinicopathologic findings of affected foals were previously described [1]. The study included n = 11 confirmed EJSCA foals (n = 9 fillies and n = 2 colts) that appeared healthy at birth but developed severe neurologic deficits within the first months of life (S1 Table). A total of n = 7 EJSCA affected foals underwent whole-genome sequencing (S1 Table). For RNA-sequencing, spinal cord tissue from n = 5 affected EJSCA foals was profiled (all fillies, median age 30 days, range 14–30 days, S1 Table). For RT-PCR of spinal cord, liver and gluteal muscle, a subset of n = 3 affected EJSCA foals were used (S1 Table). For Western blot analysis, spinal cord, liver, and gluteal muscle from n = 3 affected foals, and cardiac muscle from n = 1 affected foal were used (S1 Table).

For the genetic investigation, blood samples were provided on all the affected foals’ dams, which were neurologically normal, of which n = 3 were used for whole-genome sequencing, along with n = 1 sire, n = 3 unaffected half-siblings and n = 1 unaffected full-sibling (S1 Table). Siblings had lived past six months of age with no evidence of ataxia. Publicly available whole-genome sequences from unaffected unrelated Quarter Horses (https://www.ebi.ac.uk/ena/data/view, SUB11328561, SUB13501748 and SUB15353402) was leveraged as an additional set of control samples. For RNA-sequencing, spinal cord tissue from n = 5 unaffected age-matched control foals was profiled (2 fillies, 3 colts, median age 180 days, range 21–2190 days, S1 Table). For RT-PCR of spinal cord and gluteal muscle, a subset of n = 3 control foals were used. For RT-PCR of liver, a subset of n = 2 control foals were used. For Western blot analysis, spinal cord and gluteal muscle from n = 3 control foals, liver from n = 2 control foals and myocardium from n = 1 control foal were used (S1 Table).

### Blood DNA extraction

Genomic DNA was isolated from whole blood samples according to the WIZARD Blood DNA Extraction Kit protocol (Promega, Madison, WI). Sample DNA concentrations were measured using QIAxpert (QIAGEN, Hilden, Germany).

### Whole-genome sequencing

Whole-genome sequencing was performed on 15 horses on the Illumina HiSeq4500 platform using paired-end, 2 x 150 bp chemistry. Average coverage depth was 30x across the Equus Caballus genome. Quality control (FastQC) indicated that % of bases possessed a Phred score > 30. Reads were mapped to the EquCab3.0 equine reference sequence [38] using BWA for Illumina mapping program [39]. Mapping quality was assessed using Samtools Flagstat [40]. SNP, INDEL discovery, and genotyping across all samples was performed for the discovery of a variant using Genome Analysis Toolkit (GATK, v4.2) [41] Variant calling was performed using GATK’s HaplotypeCaller to generate genomic variant call format (gVCF) files for each sample. Joint genotyping was conducted with GenotypeGVCFs to create a multi-sample VCF. The resulting VCF was filtered using SNPSift [23] to filter the resulting.vcf file by quality, using a variant Phred threshold of 30 (Q ≥ 30). Whole genome sequences were deposited in the NCBI Sequence Read Archive (https://ncbi.nlm.nih.gov/subs/sra/) (PRJNA1395672). For publicly available files, fastq files were obtained from the SRA archive (https://www.ebi.ac.uk/ena/data/view, SRR19364590, SRR28735411 and SRR34301010) and trimmed for quality.

A whole-genome genome-wide association study (WGS-GWAS) was performed using n = 7 EJSCA foals, n = 4 parents (coded as unknown) and n = 32 control Quarter Horses. Case/control status was assigned using SNPSift caseControl [42], followed by filtering based on an allelic *P* value of 1 x 10^-11^. In addition to assessing significantly associated variants called with FreeBayes, raw bam files were visually inspected using Integrative Genome Viewer [43] in the candidate regions for any structural variants, including duplications, inversions, and large deletions or insertions. SNPEff was then used to predict the effect on protein function of the alternate variants, classifying variants as having ‘HIGH’, ‘MODERATE’, ‘LOW’, or ‘MODIFIER’ putative effects [42]. Chromosomal distribution of the resulting significantly associated variants (*P* < 1 x 10^-11^) was assessed. Variants with predicted ‘HIGH’ or ‘MODERATE’ effects were then screened in the publicly available database of equine whole-genome sequences using NCBI’s Sequence Read Archive (SRA) database (https://www.ncbi.nlm.nih.gov/sra) and excluded if found in breeds other than the Quarter Horse. Functional effects were predicted using the Ensembl Variant Effect Predictor (https://www.ensembl.org).

### mRNA-sequencing

To quantify gene expression across the four genes of interest in the candidate region (*FADS6, FDXR, GRIN2C* and *TMEM104*), mRNA-sequencing on spinal cord tissue was performed on n = 5 EJSCA cases and n = 6 unaffected age-matched controls (S1 Table). RNA was isolated from spinal cord tissue as previously described [22] and RNA integrity scores (RIN) assessed using the BioAnalyzer High Sensitivity RNA Assay. RIN scores below 7.0 were considered to have failed quality control. Libraries were prepared using PolyA selection with the NEBNext Ultra II Directional Prep Kit. The resulting libraries were sequenced on Illumina, with 150 base pair paired end reads at a depth of 40 million reads per sample. Adapter trimming, poly A trimming, quality trimming, length filtering, and removal of PCR duplicates were conducted using HTStream, version 1.3.3 [44]. The resulting reads were aligned to EquCab.3.0 using STAR, version 2.7.10b [45]. Differential expression analyses were conducted using the limma-voom Bioconductor pipeline (limma version, edgeR version, in R version 4.2.2 (2022-10-31) [46]), using a model with effects for disease status, sex, and age. Low expressed genes were filtered prior to analysis using the function filterByExpr in edgeR, leaving 16,449 genes. Raw bam files of *FDXR* were visually inspected using Integrative Genome Viewer [43]. RNA-sequences were deposited in the NCBI Sequence Read Archive (https://ncbi.nlm.nih.gov/subs/sra/) (PRJNA1395676).

### qRT-PCR Validation of *FDXR* expression

To validate decreased *FDXR* expression in spinal cord from EJSCA affected foals, qRT-PCR was performed using a subset of animals from the mRNA-seq study (n = 3 EJSCA affected foals and n = 3 age-matched unrelated unaffected control foals, S1 Table). Primer3Plus software [47] and DNA oligonucleotides were synthesized by Integrated DNA Technologies, with primers flanking exons 3 and 4 of *FDXR* (F 5’CCCATGTGGACATCTTCGAGAA3’, R 5’ GAAGGCACAGCGGTCAGAG3’) and *Beta-actin* (*ACTB*) was used as a reference gene (F 5’AAGGAGAAGCTCTGCTATGTCG3’, R 5’GGGCAGCTCGTAGCTCTTC3’). The reverse transcription of the two-step RT-qPCR was performed using Superscript III (Invitrogen) and random hexamers with 1.5 µg total RNA. Quantitative PCR Reactions were performed in a 10 µL reaction volume using the QIAGEN Rotor-Gene SYBRGreen PCR Kit (QIAGEN, Valencia, CA, USA. Each tube contained 1:10 dilution of total converted cDNA, and 1 µm final primer concentration for each forward and reverse primer. PCR was performed on a Rotor-Gene Q 72-well thermocycler (QIAGEN, Valencia, CA, USA) as follows: 5 min at 95 °C; 35 cycles of 5 s at 95 °C and 10 s at 58 °C; melt curve ramping from 60 °C to 95 °C, rising by 1 °C at each step. Each reaction was run in triplicate and each run included a no-template control. Efficiencies for each gene were calculated at 90–110%. Relative quantitation of gene expression was calculated by comparative threshold cycle method (2 − ΔΔCt) using the Ct of the housekeeping gene with the lowest variance. Data were analyzed using a student’s T test, with significance set at p < 0.05.

### Sanger sequencing validation

To confirm the putative variants identified on whole-genome sequencing, primers were designed using Primer3Plus software [47] and DNA oligonucleotides synthesized by Integrated DNA Technologies (F 5’TGCATCCTCAAGACGACCTG3’, R 5’TTCCACCCTCATGCATTCCC3’). Amplification of products was performed using end-point PCR. Products were loaded with Invitrogen TrackIt Cyan/Orange Loading Buffer and electrophoresis was performed using a 1.5% agarose gel stained with SYBR Safe DNA Gel Stain (Invitrogen; s33102). Fragments were visualized using the Azur c150 Gel Imaging Workstation. PCR was performed using the Invitrogen Platinum *Taq* DNA Polymerase kit (10966). Each 25-µl PCR reaction comprised 0.1 ul of Platinum *Taq* DNA Polymerase, 2.5 µl of 10x PCR Buffer -Mg, 0.75 ul of 5 mM MgCl_2_, 0.5 ul of 10 mM dNTPs, 0.25 ul of 20 uM forward and reverse primers (Integrated DNA Technologies), 1 ul KB Extender, and 1 µl of 50 ng/ul genomic DNA. Standard PCR conditions were performed as follows: 94 °C for 2 min and 35 cycles of 94 °C denaturation for 30 s, 57 °C annealing for 30 s, and 72 °C extension for 1 min. Sanger sequencing was performed using the commercially available services provided by the UC Berkeley DNA Sequencing Facility (https://ucberkeleydnasequencing.com/home). Resulting sequences were aligned to EquCab3.0 (http://www.ncbi.nlm.nih.gov/genome/145) and analyzed with SEQUENCER software (Gene Codes Corp.). The n = 11 affected foals, n = 12 dams, n = 4 sires, n = 11 siblings, and n = 8 unrelated control horses were genotyped (S1 Table**)**.

### Population allele frequency

To estimate the Quarter Horse population allele frequency and test additional animals for the presence of this variant, 1,060 presumed unaffected Quarter Horses with samples banked at the UC Davis Veterinary Genetics Laboratory (VGL) were genotyped for the *FDXR* intronic variant. Samples were randomly selected from those with consent from owners or breed organizations to use for research purposes and individuals with known first degree relationships were removed. Genotyping was performed using the assay developed and now commercially available at the VGL (https://vgl.ucdavis.edu/test/equine-juvenile-spinocerebellar-ataxia-ejsca). The allele frequency for the *FDXR* variant was calculated as:


$$\begin{array}{c} f_{D}\;=\;(H_{\text{D}/\text{N}}\;+\text{ 2}H_{\text{D}/\text{D}})\;/\text{ 2n} \end{array}$$

$$\begin{array}{c} {SE}_{f\text{D}}\;=\;((f_{\text{D}}\;*\;(\text{1 }-\;f_{\text{D}}))\;/\text{ 2n}) \end{array}$$

Where:

***f***_**D**_ represents the frequency of the disease allele

***SE***_***f*D**_ represents the standard error of ***f***_**D**_

***H***_**D/N**_ represents the number of horses with one disease allele (heterozygous)

***H***_**D/D**_ represents the number of horses with two disease alleles (homozygous)

**n** represents tested population number

An additional 181 horses across 26 breeds were screened and no other carriers were identified in other breeds (S3 Table).

### *FDXR* conservation and expression

Scores were determined for each orthologous human variant using the 100-vertebrate score by phastCons (https://genome.ucsc.edu/) since conservation scores are not available in the EquCab3.0 genome browser within UCSC (https://genome.ucsc.edu/). *FDXR* expression was evaluated in tissues available from the equine FAANG initiative (https://www.ebi.ac.uk/ena/data/view/ERA1487553) [22].

### *FDXR* c.177 + 1778G > C functional validation

#### RT-PCR.

To define the boundaries of the cryptic exon identified in *FDXR*, RT-PCR was performed on spinal cord, liver and gluteal muscle of n = 3 EJSCA foals and n = 3 controls (S1 Table). Primers were designed to anneal to exon 2 and exon 5 of *FDXR*-203, as annotated in the Ensembl database for EquCab3.0 (https://useast.ensembl.org/Equus_caballus/Info/Index). Primer sequences were F 5’CTTCTACACGGCCCAACACC 3’, R 5’ GAGCCCATTGTACCAGCCC 3’. Gel extraction and Sanger sequencing of the resulting bands was unsuccessful. Therefore, long-read sequencing was performed on the resulting bands from EJSCA and control horses.

#### PromethION nanopore long-read sequencing.

Long-read PromethION Nanopore sequencing of the resulting RT-PCR products from spinal cord tissue was performed in n = 2 EJSCA foals and n = 4 controls (S1 Table). Barcoding PCR was performed using the PBC001 kit, barcoded samples were pooled and the sequencing library was prepared using LSK114 kit. The libraries were sequenced on R10.4.1 PromethION flow cell. Data was base called in the super accurate mode using dorado v4.3.0, 400 bps. The yield was 12M reads with more than 1M reads per barcode. Only data >Q10 was used for downstream analysis. For analysis, reads were randomly subsampled at 100,000 reads per sample using seqtk [48] and primer sequences trimmed using a custom Python script. Reads within an inexact primer match were omitted from the analysis (S5 Table). Samples were then merged into a single file and the clustering algorithm CD-HIT [49] was used to cluster reads that were 98% similar to each other based on global sequence identify. Significant clusters were defined as those that had > 10% of at least one sample’s total reads in them. With the resulting four clusters, representing 82.5% of all analyzed reads, 25 sequences from each cluster were again randomly subsampled and ClustalW [50] was used to identify a consensus sequence using default parameters. Sequences were aligned to EquCab3.0 using BLAST.

#### Western blotting.

For total protein extraction, 10–20 mg of gluteal muscle, liver, and heart and 30–40 mg of spinal cord was used. Tissues were transferred to 1.5 mL microcentrifuge tubes on ice, 600 uL RIPA lysis buffer was added, and tissues were homogenized with plastic pestles. Contents were agitated on a plate shaker for 2 h at 4°C, centrifuged at 13,000 x g for 20 minutes at 4°C, and supernatant was collected. Isolated protein sample concentrations were quantified using the Pierce BCA Protein Assay Kit (Thermo Scientific; Cat no. 23225) and samples were stored at -80°C. All protein concentrations were diluted to 1 ug/1 ul with 1x Tris Glycine SDS and 15 ul lysate samples mixed with 7.13 ul 4x Laemmli sample buffer (Bio-Rad; Cat no. 1610747) and 0.75 ul 2-Mercaptoethanol (Bio-Rad; Cat no. 1610710). Samples were heated for 5 minutes at 90°C. SDS-PAGE and blotting was performed using the Bio-Rad Mini-PROTEAN Tetra cell, 10% Mini-PROTEAN TGX Precast Protein Gels, 10-well, 30 µl (Cat no. 4561033), and Bio-Rad Nitrocellulose Membranes, Precut, 0.2 µm, 7 x 8.4 cm (Cat no.16201460). 15 ug total protein was loaded for each sample. Biological replicates included n = 3 controls and n = 3 cases (spinal cord) and n = 2 controls and n = 3 cases (liver) (S1A, S1B, S1C, and S1D Fig).

To detect FDXR protein, a polyclonal rabbit IgG primary antibody (Novus Biologicals NBP2–38706) was used at a dilution ratio of 1/10000, and a goat anti-rabbit HRP-linked secondary antibody (Cell Signaling Technology 7074P2) was used at a dilution ratio of 1/10000. To detect actin, a mouse monoclonal IgG1 κ beta Actin (C4) primary antibody (Santa Cruz Biotechnology sc-47778) was used at a dilution ratio of 1/10000 and a goat anti-mouse IgG (H + L) linked cross-adsorbed HRP-linked secondary antibody (Invitrogen A16072) was used at a dilution ratio of 1/5000. Imaging was performed using the Bio-Techne ProteinSimple FluorChem E System according to the manufacturer’s instructions.

In the initial set of Western Blots (S1A, S1B, S1C, and S1E Fig), both human colon cell lysate and mouse lung lyase were used as positive controls, since human is the target species of the rabbit polyclonal anti-FDXR antibody (Novus NBP2‑38706) with confirmed reactivity at ~54 kDa. Mouse lung lyase produced a band at~54 kDa and therefore this antibody recognized both human and murine FDXR under our Western blot conditions and was subsequently included in all experiments as a positive control. Alignment of the FDXR immunogen peptide region to the predicted equine FDXR sequence shows 94.6% amino acid identity with the antibody epitope and alignment was 89.2% for the predicted murine FDXR protein sequence [51]. This indicates that mouse and horse FDXR are sufficiently conserved at the epitope level for reliable cross-species detection.

## Supporting information

S1 Fig Western blotting of FDXR in additional cases and controls. (DOCX)

S1 Table Overall demographics of horses used for the whole-genome sequencing and subsequent functional validation. MC = male castrated, FS = female spayed, SC = spinal cord, GM = gluteal muscle, LIV = liver, HRT = myocardium.
(XLSX)

S2 Table Partial vcf file and public dataset searches of 77 variants associated with EJSCA at at p < 1x10^-11^. (XLSX)

S3 Table Summary of breeds screened for the EJSCA mutation and corresponding genotype and estimated allele frequencies. (XLSX)

S4 Table Clusters identified by cDNA Nanopore long-read sequencing in control and EJSCA horses. (XLSX)

S5 Table Number of reads used for analysis after randomly subsampling 100,000 reads and filtering out inexact primer regions. (XLSX)
