# Supplementary material for: An intronic variant in Ferredoxin Reductase (FDXR) creates a cryptic exon in Quarter Horses with Equine Juvenile Spinocerebellar Ataxia
Source: PLoS Genet. 2026 May 20;22(5):e1012158. doi: 10.1371/journal.pgen.1012158 (PMC13215600; doi:10.1371/journal.pgen.1012158)
Supplement: S1 Fig — (DOCX) [file pgen.1012158.s001.docx]

**S1 Fig A**

**Original Image of Spinal Cord Biologic Replicates FDXR (Fig. 4A: n=3 EJSCA vs. n=3 Control)**

Novus Rabbit anti-FDXR Primary Ab (Novus Biologicals NBP2-38706) | Anti-rabbit HRP (Cell Signaling Technology 7074P2) | Spinal Cord

**
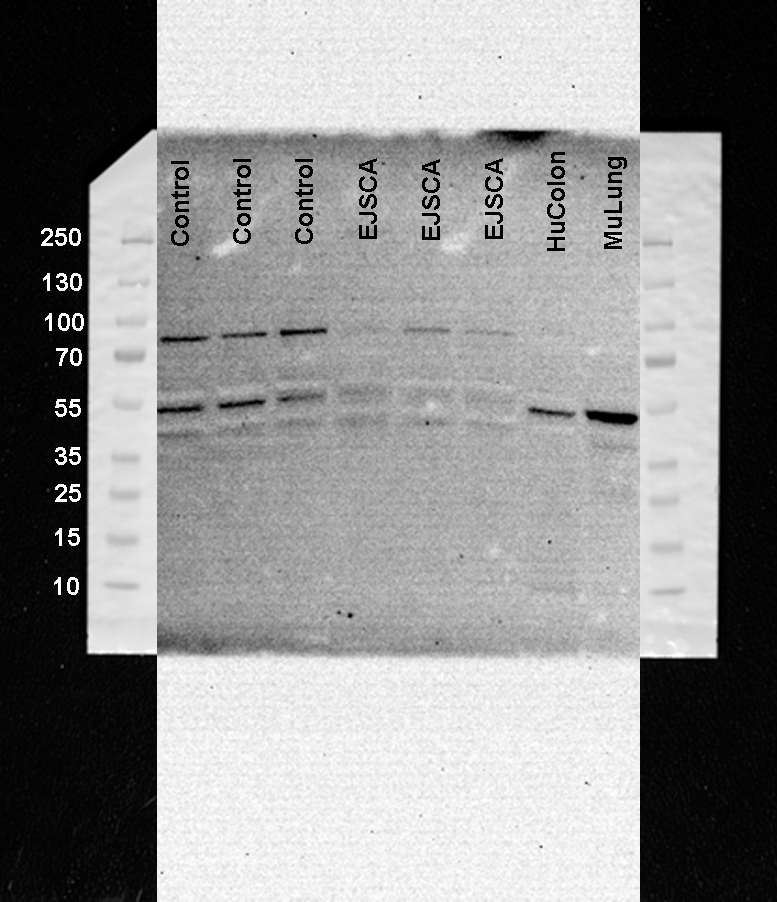
**

**S1 Fig B**

**Spinal Cord Biologic Replicates Actin (Fig. 4A: n=3 EJSCA vs. n=3 Control)**

Mouse anti-Actin Primary (Santa Cruz Biotechnology SC-56459) | anti-Mouse HRP Secondary (Invitrogen A16072) | Spinal Cord


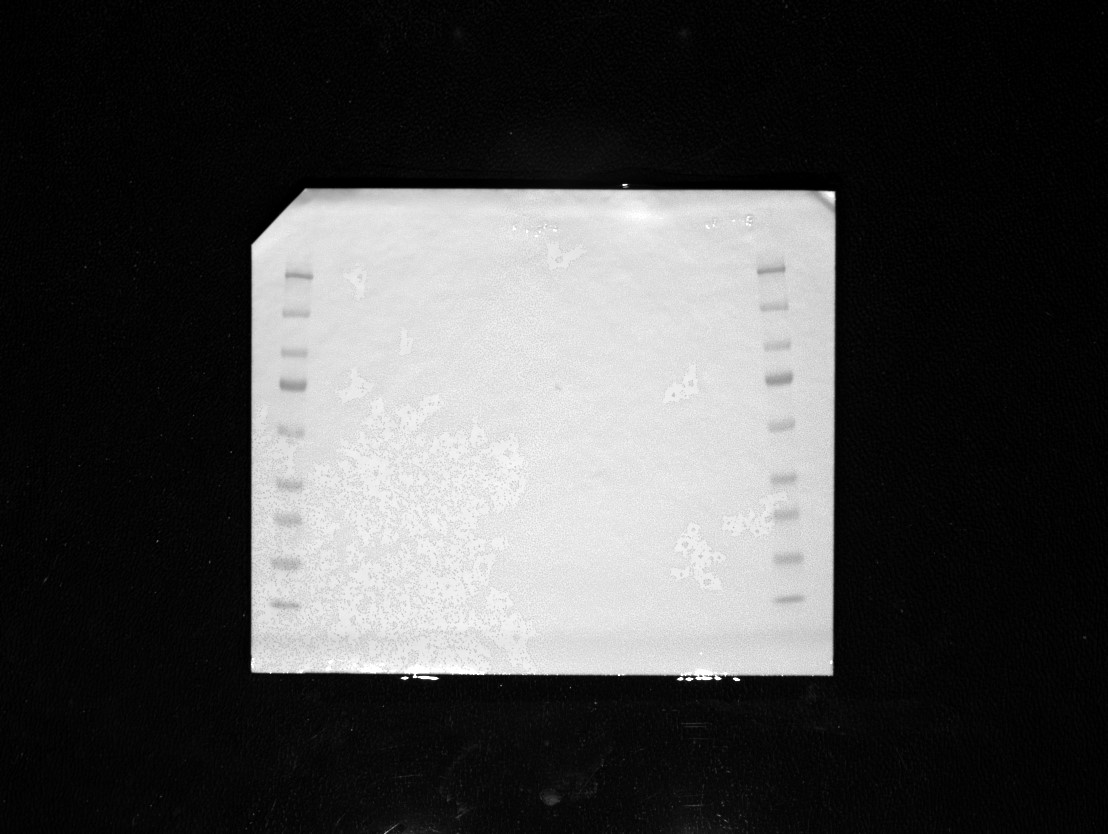

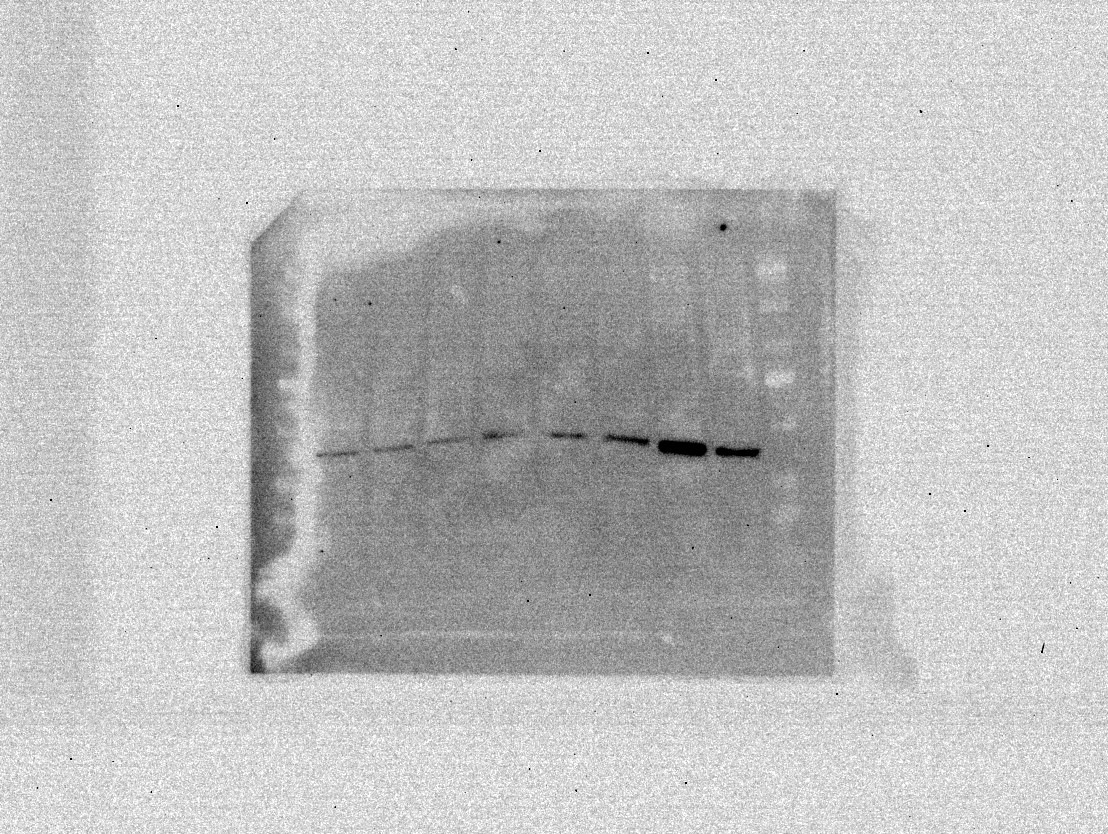


**MuLung**

**Control**

**Control**

**Control**

**EJSCA Case**

**EJSCA Case**

**EJSCA Case**

**HuColon**

**S1 Fig C**

**Original WB images for Figure 4c (Spinal cord, Liver, Gluteal)**

Novus Rabbit anti-FDXR Primary Ab (Novus Biologicals NBP2-38706) | Anti-rabbit HRP (Cell Signaling Technology 7074P2) | Spinal Cord


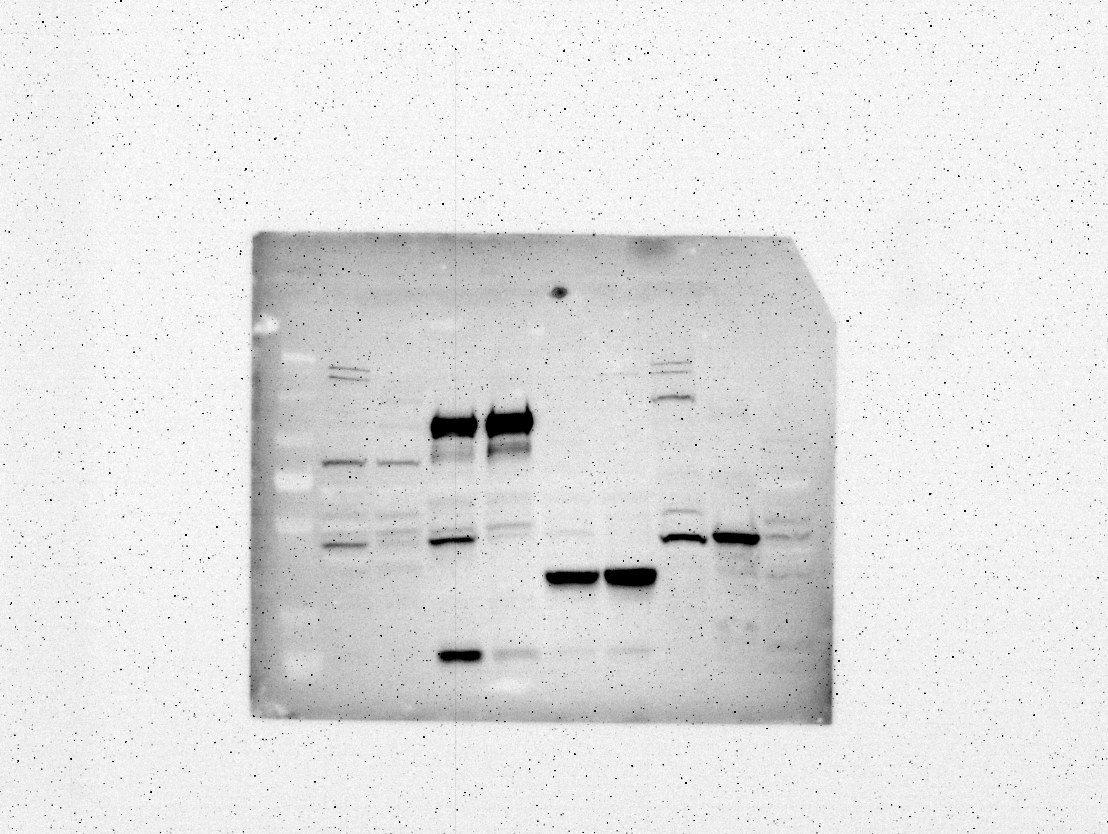


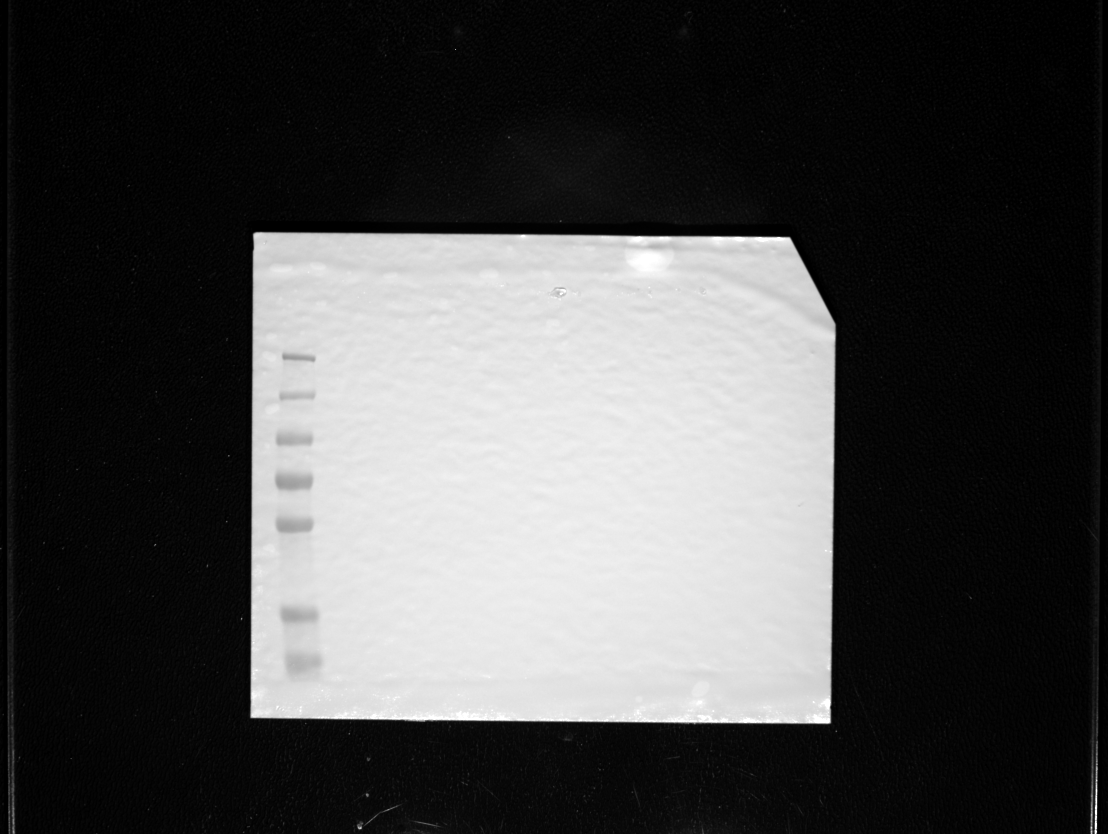


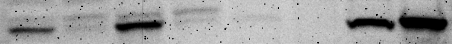


Control SC

EJSCA SC

Control LIV

EJSCA LIV

Control GM

EJSCA GM

Mouse Lung

Human Colon

**S1 Fig D**

**Original WB images for Figure 4c (Spinal cord, Liver, Heart, Gluteal)**

Novus Rabbit anti-FDXR Primary Ab (Novus Biologicals NBP2-38706) | Anti-rabbit HRP (Cell Signaling Technology 7074P2) | Spinal Cord


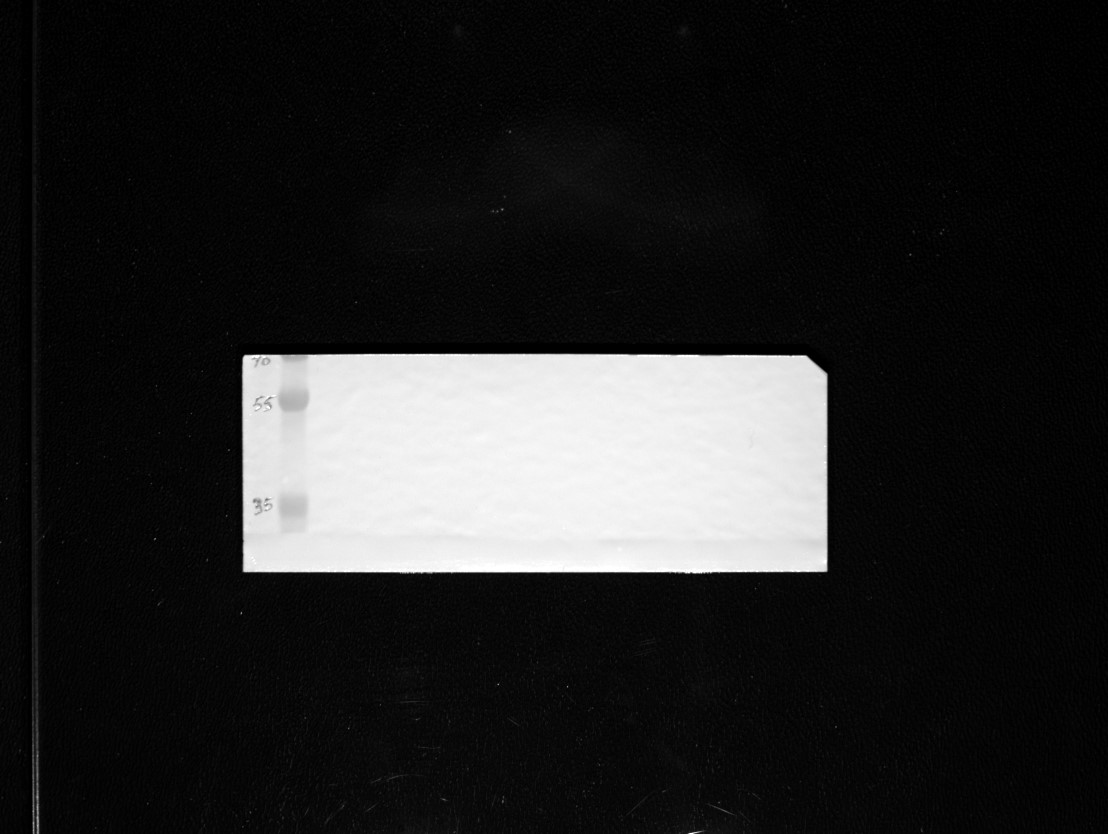


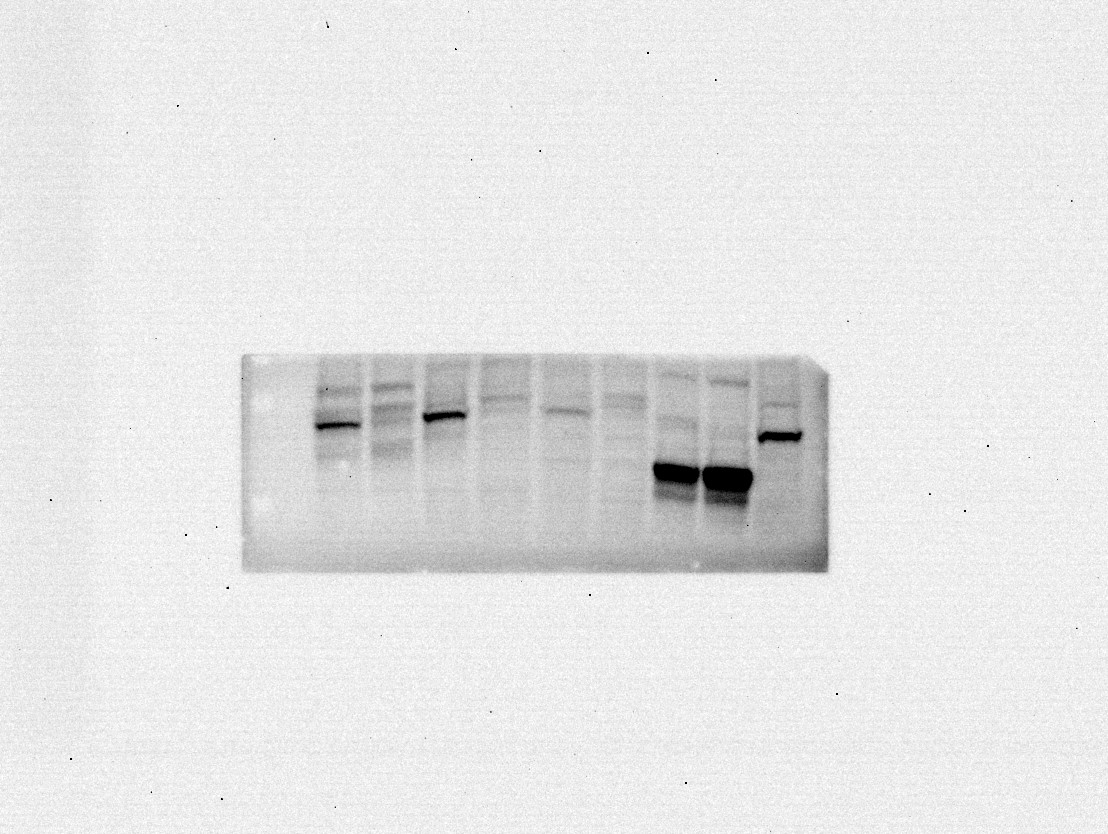


Control SC

EJSCA SC

Control LIV

EJSCA LIV

Control HRT

EJSCA HRT

Control GM

EJSCA GM

Mouse Lung


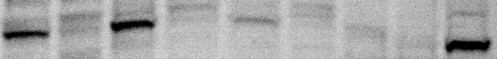


**S1 Fig E**

**Beta-actin WB Original Image (Fig. 4c)**

**Mouse B-Actin (C4) Primary (Santa Cruz Biotechnology sc-47778) | anti-Mouse HRP Secondary (Invitrogen A16072) | SC, LIV, HRT and GM**


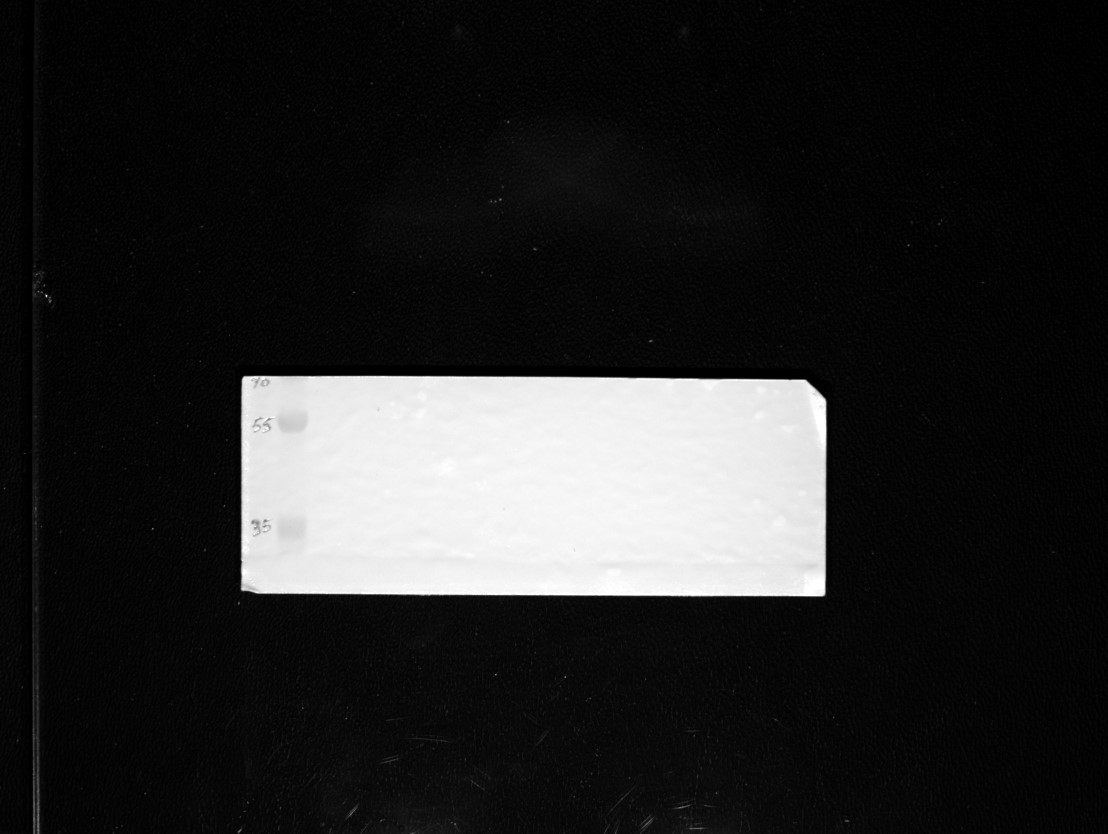

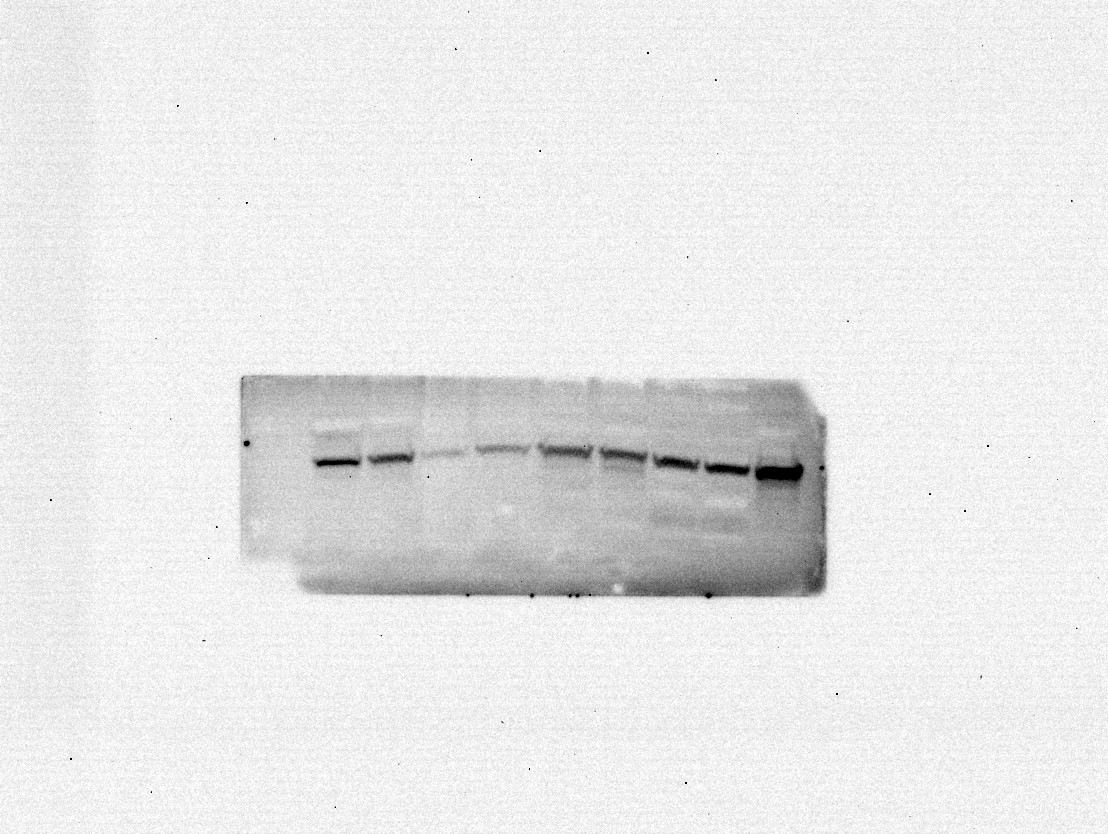


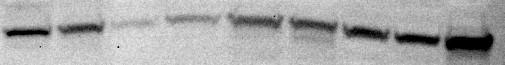


Control SC

Case SC

Control LIV

Case LIV

Control HRT

Case HRT

Control GM

Case GM

Mouse Lung

Human Colon


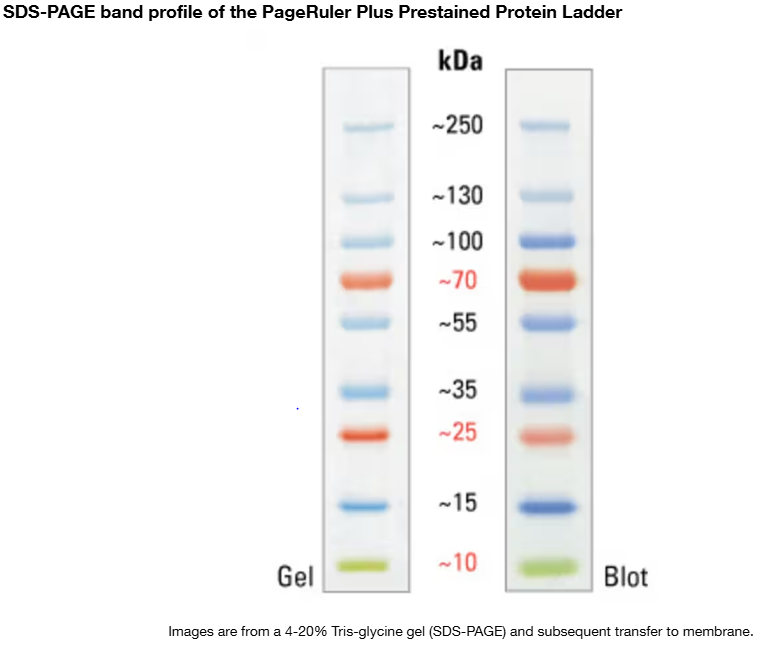
**S1 Fig F**
